# Colonoscopy surveillance after colorectal cancer surgery: meta-analysis

**DOI:** 10.1093/bjsopen/zrag117

**Published:** 2026-11-04

**Authors:** Joni Panula, Kalle E Hokkanen, Toni T Seppälä

**Affiliations:** Department of Gastroenterology and Alimentary Tract Surgery and Tays Cancer Centre, Tampere University Hospital, Wellbeing Services County of Pirkanmaa, Tampere, Finland; Center for Cancer Eradication Research, Faculty of Medicine and Health Technology, Tampere University, Tampere, Finland; Center for Cancer Eradication Research, Faculty of Medicine and Health Technology, Tampere University, Tampere, Finland; Department of Gastroenterology and Alimentary Tract Surgery and Tays Cancer Centre, Tampere University Hospital, Wellbeing Services County of Pirkanmaa, Tampere, Finland; Center for Cancer Eradication Research, Faculty of Medicine and Health Technology, Tampere University, Tampere, Finland; Applied Tumor Genomics, Research Programs Unit, University of Helsinki, Helsinki, Finland; Abdominal Center, Helsinki University Central Hospital, HUS, Helsinki, Finland

**Keywords:** colorectal neoplasms, postoperative follow-up, cancer recurrence, survival, systematic review

## Abstract

**Background:**

Colonoscopy surveillance is recommended following colorectal cancer (CRC) surgery, yet the evidence supporting the recommendations has major quality issues. This study assessed the independent impact of colonoscopy surveillance within 5 years after CRC surgery.

**Methods:**

A systematic review and meta-analysis were conducted. Medline, Scopus, Web of Science, and the Cochrane Central Register of Controlled Trials were searched from inception until 18 June 2026 for randomized clinical trials comparing follow-up protocols including colonoscopy surveillance (intervention) to those without colonoscopy surveillance (control) within 5 years after CRC surgery. Risk of bias was assessed using the RoB 2 tool. Random-effects meta-analyses were conducted to estimate the strength of associations with recurrence detection, reoperation rate, and survival. The number needed to scope (NNS) to detect one recurrence and health economic considerations were estimated. The study protocol has been registered in PROSPERO.

**Results:**

Of 4381 records, five trials with 2337 patients were included. Four trials enrolled patients between 1983 and 1994, predating subsequent advances in CRC treatment. All trials used additional surveillance modalities, leading to a high risk of bias. Follow-up protocols with colonoscopy were not associated with recurrence profile, with risk ratios (RRs) of 0.74 (95% confidence interval (c.i.) 0.32 to 1.69), 1.23 (95% c.i. 0.93 to 1.63), and 0.93 (95% c.i. 0.76 to 1.14) for intraluminal, locoregional, and distant recurrence, respectively. Colonoscopy during follow-up was associated with a higher rate of asymptomatic recurrence (RR 2.38; 95% c.i. 1.53 to 3.69). More curative-intent reoperations were performed in the intervention than control group (RR 1.66; 95% c.i. 1.16 to 2.37), but this was not translated into improved survival (hazard ratio 0.92; 95% c.i. 0.77 to 1.11). The NNS was 159 (95% c.i. 101 to 267), resulting in estimated cost per colonoscopy-detected recurrence of €104 100 (95% c.i. €66 200 to 174 900).

**Conclusion:**

Current evidence from randomized trials does not clearly demonstrate that postoperative colonoscopy surveillance improves survival, although interpretation is limited by the age of the trials and confounding from concurrent surveillance modalities. Contemporary randomized trials are required to better define the independent contribution of colonoscopy within modern multimodal surveillance pathways.

## Teaser

This systematic review and meta-analysis of randomized clinical trials evaluated the impact of colonoscopy-based follow-up within 5 years after colorectal cancer resection. Colonoscopy was associated with increased detection of asymptomatic recurrences and higher rates of curative-intent reoperations, but did not improve overall survival or alter recurrence patterns. The high number needed to scope and substantial costs per detected recurrence raise concerns about the clinical and economic value of the routine use of colonoscopy in this setting.

## Introduction

Colonoscopy surveillance has been one of the cornerstones of follow-up after colorectal cancer (CRC) surgery. Current guidelines recommend repeated examinations over several years^1–3^ based on the interpretation that colonoscopy efficiently detects intraluminal recurrences and metachronous cancers at an earlier stage, translating into improved survival. The evidence supporting this practice has been largely unchallenged^4^, although it mostly dates back to an era with a greater than 20% risk of local recurrence after rectal cancer^5^. Postoperative follow-up represents a substantial use of healthcare resources, with mean costs ranging from approximately €3700 to 7900 depending on the surveillance intensity^6^. Given the cost-effective alternative spending priorities, such as CRC screening^7^, efficient allocation of resources requires that postoperative surveillance is based on evidence of clinical benefit.

Most recurrences occur at distant sites (15–20%) beyond the reach of colonoscopy, whereas locoregional (2–5%) and intraluminal (< 1%) recurrences have become relatively uncommon^8–10^ and are often symptomatic^11^. As a result, the yield of routine colonoscopy surveillance in the era of modern surgical and oncological treatments is likely to be low and its impact on survival remains uncertain. At the same time, patients bear the burdens of repeated bowel preparations, procedural risks, and psychological stress, and there is constant pressure to optimize the use of endoscopy resources for the most high-yield indications. Despite these limitations, colonoscopy retains a central role in follow-up protocols, embedded in clinical practice as if its value were self-evident.

Although previous meta-analyses have examined follow-up after CRC surgery^12,13^, they lack critical and comprehensive assessment of the role of colonoscopy specifically. In this context, a focused meta-analysis is needed to evaluate whether colonoscopy surveillance provides meaningful clinical benefits, or whether its widespread use persists more from convention than evidence.

## Methods

### Study design and systematic review registration

A systematic review and meta-analysis of the literature was performed following the instructions outlined in the PRISMA guidelines^14^. A predefined study protocol was registered on PROSPERO (CRD4202511118699).

Because this study is based on aggregated data from published reports, ethics approval was not required.

### Eligibility criteria

Eligible randomized clinical trials (RCTs) compared patients aged ≥ 18 years who underwent a follow-up protocol with routine colonoscopy surveillance to those who underwent a follow-up without routine colonoscopies within 5 years after curative-intent CRC resection. The 5-year time point was chosen to match the recommended follow-up duration^1–3^. Because real-world surveillance protocols involve multiple modalities^2^ that may be investigated concurrently, studies evaluating concurrent surveillance modalities other than colonoscopy were included. Trials comparing different intensities of colonoscopy surveillance or involving local excision techniques as index surgery were excluded. No language limitations were applied.

### Search strategy

PubMed (Medline), Scopus, Web of Science, and the Cochrane Central Register of Controlled Trials (CENTRAL) were systematically searched from inception to 18 June 2026. The search consisted of three blocks of search terms for the concepts of CRC, postoperative colonoscopy surveillance, and RCT. The search strategy is detailed in *Table S1*. In addition, the reference lists of other relevant systematic reviews were assessed.

### Study selection

Using standard, pilot-tested forms, two authors (J.P. and K.E.H.) independently screened all titles and abstracts identified by the literature search. The reviewers evaluated the full texts identified as potentially eligible by either reviewer for eligibility. Any disagreements were resolved by discussion or, if necessary, by consulting with a third author (T.T.S.).

### Data analysis

Data were extracted independently by J.P. and checked by T.T.S. using a standard, pilot-tested form. The following baseline characteristics were extracted: study characteristics (enrolment period, country, length of follow-up), baseline characteristics of the included patients (sample size, age, sex, cancer localization, and cancer stage), and intervention (frequency and total number of colonoscopies and additional surveillance modalities). Overall survival was the main outcome of interest. The following were secondary outcome measures: recurrences (intraluminal, locoregional, distant, and asymptomatic), metachronous cancers, time to recurrence, curative-intent reoperations (total and bowel reresections), and cancer-specific survival. Intraluminal recurrence was defined as occurring at or near the anastomosis, and locoregional recurrence was defined as occurring at the operative field with possible, but not necessarily, extension into the bowel lumen and included intraluminal recurrences. Recurrences with simultaneous local and distal spread were classified as distant recurrences.

### Risk of bias assessment

The risk of bias was assessed by J.P. and checked by T.T.S. using the revised Cochrane RoB 2 tool^15^ for assessing risk of bias in randomized trials according to five domains: randomization process, deviations from intended interventions, missing outcome data, measurement of the outcome, and selection of the reported result. For each domain, trials were judged to be either at low risk of bias, to have some concerns, or at high risk of bias.

### Data synthesis and statistical analysis

The effect measure of overall mortality was assessed using hazard ratios (HRs). To estimate HRs, when they were not reported, a validated algorithm was applied to reconstruct individual patient data from published Kaplan–Meier curves^16^. Time to recurrence was reported as the mean difference (MD). If the mean was not reported, it was converted from the median using the Hozo method^17^ or computed from reconstructed individual patient data. Remaining outcomes were measured using relative risk (RR). Pooled estimates were presented in forest plots and calculated by a random-effects model using the DerSimonian–Laird method^18^. Heterogeneity was evaluated using Cochran's Q test, the *I*^2^ statistic, and τ^2^. Publication bias was planned to be assessed using funnel plots and Egger's test when ten or more studies were available for any outcome as specified in the study protocol. However, because only five studies were included, these tests were not performed because test power was considered too low to distinguish chance from real asymmetry^19^. For the intervention group, the number needed to scope (NNS) to detect one recurrence and the cost per quality-adjusted life-year (QALY) were estimated. The NNS was calculated by dividing the total number of colonoscopies by the number of colonoscopy-detected recurrences. The cost per QALY was calculated by dividing the cost per colonoscopy–detected recurrence by the estimated QALYs gained.

All statistical analyses and data visualizations were conducted using R version 4.4.2 (R Foundation for Statistical Computing, Vienna, Austria). Forest plots were generated using the metafor package^20^, individual patient data were reconstructed using the IPDfromKM package^21^, and HRs were generated using the survival package^22^.

### Certainty of evidence assessment

Certainty of evidence was assessed according to the GRADE approach^23^. The GRADE assessment was limited to the critical outcomes for colonoscopy surveillance: intraluminal recurrence, locoregional recurrence, asymptomatic recurrence, curative-intent reoperations, and overall mortality.

### Amendments to the protocol

Amendments to the protocol were made after data extraction began. Specifically, the outcome time to reoperation was not reported consistently across the studies. Therefore, time to recurrence was examined instead.

### Sex and gender analysis

Sex- and gender-disaggregated analyses were not possible because the included studies did not consistently report outcomes stratified by sex or gender, and individual participant data were unavailable.

### Patient and public involvement

Patients and members of the public were not involved in the design, conduct, reporting, or dissemination plans of this research.

## Results

### Study selection

In all, 4381 records were identified through the database search. After the removal of 2063 duplicates and the screening of the 2318 remaining titles and abstracts, 103 full-text articles were assessed for eligibility. Because the objective of this study was to isolate the effect of colonoscopy, four studies comparing higher-intensity to lower-intensity colonoscopy surveillance^24–27^ (rather than its presence *versus* absence) and one study investigating the effect of rigid sigmoidoscopy^28^ were excluded. Five unique studies, reported in six publications, met all inclusion criteria and were included in the review^29–34^ (*Fig. 1*).

**Figure 1:**
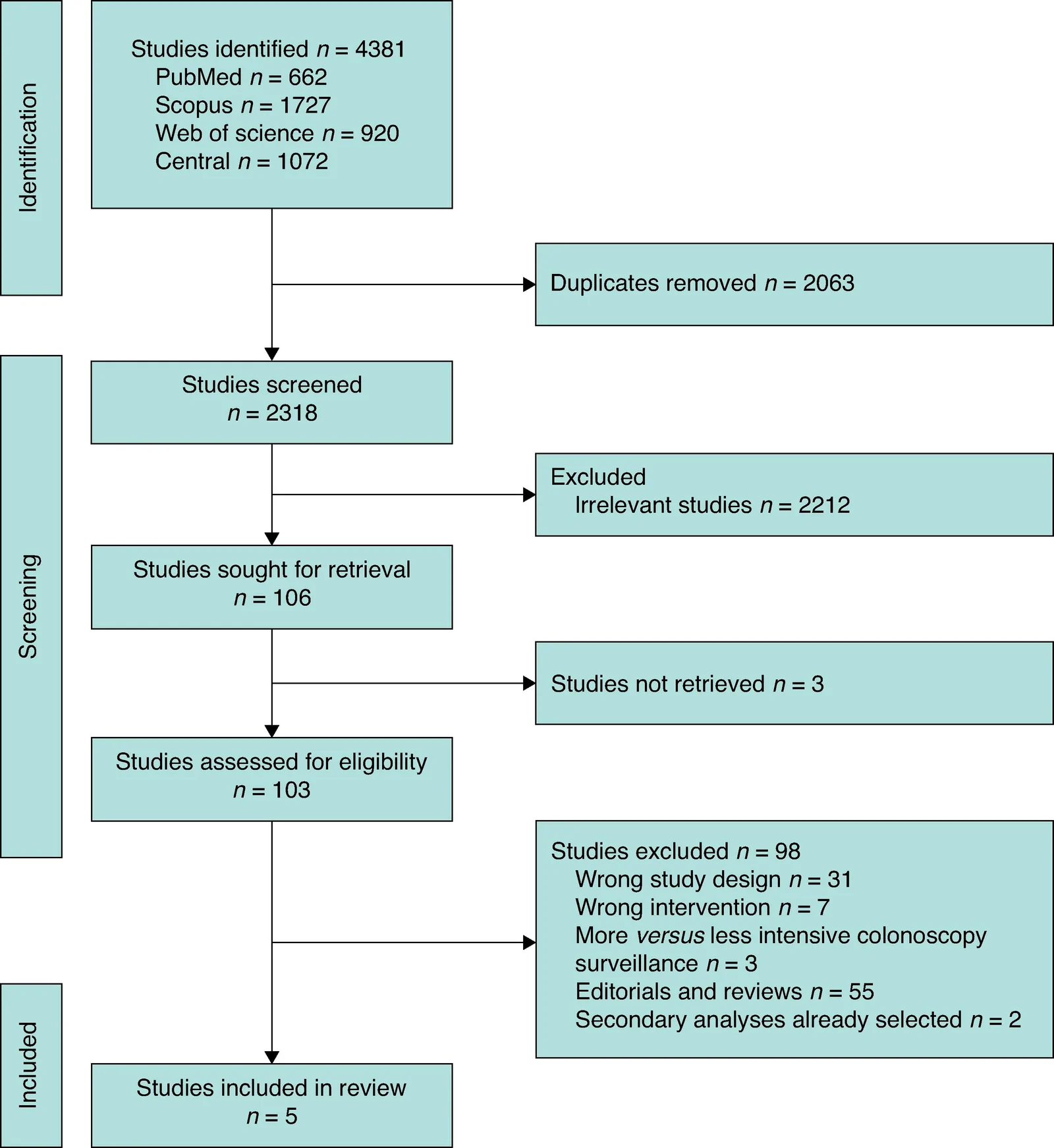
PRISMA flow diagram illustrating the selection process CENTRAL, Cochrane Central Register of Controlled Trials.

### Study characteristics

The five included RCTs were published between 1995 and 2014, collectively enrolling 2337 patients (1163 in the intervention group and 1174 in the control group)^29–34^. Four trials enrolled patients between 1983 and 1994^29–33^, whereas the most recent trial recruited patients between 2003 and 2009^34^. Patients in the intervention group underwent between one and eight scheduled colonoscopies per patient during the first 5 years after surgery. All trials investigated additional surveillance modalities concurrently with colonoscopy. Study characteristics are summarized in *Table 1* and detailed surveillance protocols for each trial are provided in *Table S2*.

**Table 1:** Characteristics of the included studies

Study	Enrolment period	Sample size	Female sex	Mean age (years)	Rectal cancer (% of cases)	Dukes’ classification			Colonoscopy surveillance	Other relevant modalities
						A	B	C		
Mäkelä *et al*.^29^	1988–1990	106	50.9%	66.0	29.2%	26.4%	45.3%	28.3%	Once a year	Liver US, abdominal CT
Ohlsson *et al*.^30^	1983–1986	107	52.3%	65.6	33.6%	17.8%	43.9%	38.3%	Alternating complete colonoscopy and endoscopic control of the anastomosis twice a year for 2 years, then once a year for the next 3 years	CEA, FOBT, CXR, pelvic CT (after APR)
Kjeldsen *et al*.^31,32^	1983–1994	597	45.4%	64.2	47.4%	23.1%	49.1%	27.8%	Twice a year for 3 years, then once a year for the next 2 years	DRE, FOBT, CXR
Schoemaker *et al*.^33^	1984–1990	325	36.3%	68.0	26.8%	21.8%	47.1%	31.1%	Once a year	CXR, liver CT
Primrose *et al*.^34^	2003–2009	1202	38.8%	69.2	30.7%	21.9%	47.6%	30.5%	At 2 years	Chest and abdominal CT

US, ultrasound; CT, computed tomography; CEA, carcinoembryonic antigen; FOBT, faecal occult blood test; CXR, chest X-ray; APR, abdominoperineal resection; DRE, digital rectal examination.

### Risk of bias in studies

Risk of bias was assessed separately for each outcome following RoB 2 guidance^15^. Because the risk of bias judgement was identical for most outcomes, a grouped assessment is presented, and exceptions are reported explicitly. All studies appeared to generate the randomization sequence adequately. However, one study^33^ likely failed to conceal allocation, resulting in high risk of bias, and three other studies^29–32^ provided insufficient information on concealment, raising some concerns (Domain 1: randomization process). Regarding bias related to the independent effect of colonoscopy surveillance, all trials included additional protocolized surveillance modalities considered as unbalanced deviations from the intended intervention. Two trials^30–32^ assessed faecal occult blood tests, with potential to detect intraluminal recurrences. Two trials^29,34^ incorporated abdominal computed tomography (CT) scans and one study^30^ evaluated carcinoembryonic antigen to facilitate the detection of intraluminal, locoregional, and distant recurrences. One trial^33^ incorporated liver CT and chest X-ray, potentially improving the detection of distant recurrences, and thereby the reoperation rate and mortality. Therefore, this domain (Domain 2: bias due to deviations from intended intervention) is judged as at high risk of bias for all studies and for most outcomes. Liver CT and chest X-ray are unlikely to affect the detection of locoregional or intraluminal recurrence or metachronous cancer, but the lack of reporting on intention-to-treat analysis raises some concerns for these outcomes in this domain in one trial^33^. There were no major concerns regarding missing outcome data (Domain 3) or measurement of outcome (Domain 4). All studies most probably reported their results adequately, although only one trial^34^ reported its prespecified study protocol (Domain 5: selection of the reported results). In summary, all trials were judged to have a high overall risk of bias for the aim of this review. *Table S3* presents grouped risk of bias assessment, noting the exception discussed above, and *Table S4* presents detailed risk of bias assessment.

### Recurrences and metachronous cancers

All trials provided data on locoregional and distant recurrence rates. Three studies^29,30,33^ reported intraluminal recurrences separately. In all, 178 locoregional recurrences were detected, leading to a mean(standard deviation (s.d.)) rate of 7.6(5.4)%, ranging from 3.4% to 21.7%, with considerable heterogeneity between studies and higher rates observed in older studies. In contrast, no such temporal association in distant recurrence rates was observed (mean(s.d.) 14.1(1.7)%, range 13.1–18.9%; *Table S5*). Surveillance protocols that included colonoscopy surveillance did not have impact on recurrence patterns: the pooled RR of detection was 1.03 (95% confidence interval (c.i.) 0.87 to 1.21) in total, 0.93 (95% c.i. 0.76 to 1.14) for distant recurrence, 1.23 (95% c.i. 0.93 to 1.63) for locoregional recurrence, and 0.74 (95% c.i. 0.32 to 1.69) for intraluminal recurrence (*Fig. 2a,b*). Four trials^29–32,34^ reported symptom status at the time of overall recurrence detection, whereas Schoemaker *et al.*^33^ reported combined symptom and screening test status. Recurrences were diagnosed more often in an asymptomatic state in the intervention group (RR 2.38; 95% c.i. 1.53 to 3.69; *Fig. 2c*). In a sensitivity analysis, omitting Schoemaker *et al.*^33^, this association remained statistically significant (RR 2.16; 95% c.i. 1.45 to 3.21; *Fig. S1*). Four studies^29–33^ reported metachronous cancer rates for both groups, and one study^34^ reported a total of three metachronous cancers. In all, 17 metachronous cancers were detected, resulting in a mean(s.d.) rate of 0.7(0.2)%, range from 0.2% to 1.7% (*Table S5*). Pooled analysis showed no significant difference in metachronous cancer rates between the groups (RR 1.76; 95% c.i. 0.61 to 5.10; *Fig. S2*).

**Figure 2:**
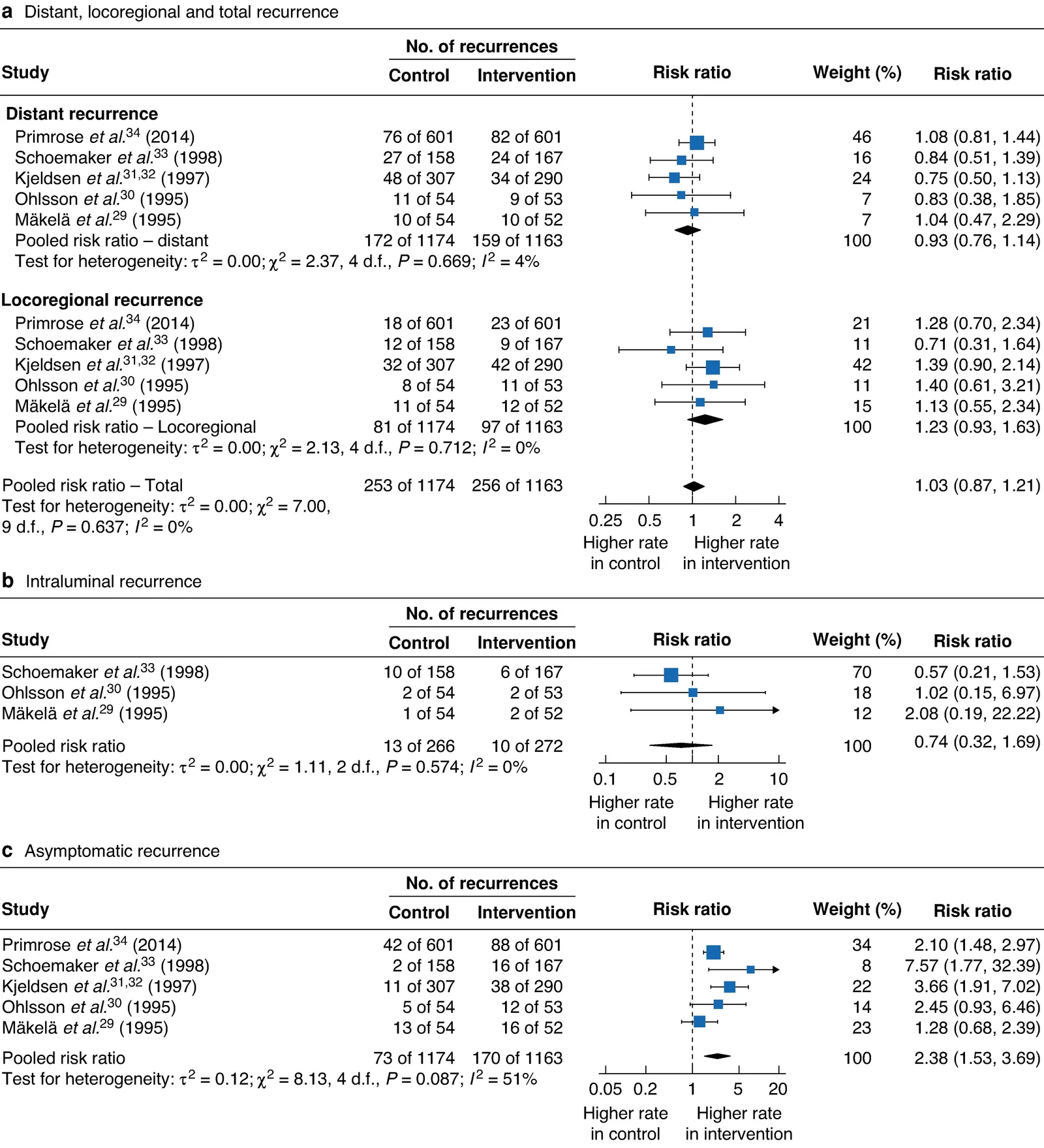
Recurrence patterns and detection of asymptomatic recurrence during follow-up with protocols either including colonoscopy (intervention) or not (control) **a** Effects of the intervention on locoregional, distant, and corresponding total recurrence. **b** Effects of the intervention on intraluminal recurrence. **c** Effects of the intervention on asymptomatic recurrence. Values in parentheses are 95% confidence intervals. Effect sizes are presented as risk ratios using a random-effects model. Square size indicates weight. The numbers of recurrences in each group are shown, along with group size.

### Yield and efficiency of colonoscopy surveillance

Among 1163 patients in the intervention group, colonoscopy detected 18 of 104 locoregional recurrences, corresponding to a detection proportion of 18.6% (95% c.i. 11.4% to 27.7%) and a colonoscopy-detected recurrence rate of 1.6% (95% c.i. 0.9% to 2.4%). In all, 2856 colonoscopies were conducted in the intervention group, translating into an NNS of 159 (95% c.i. 101 to 267) to detect one recurrence. The surveillance modalities detecting metachronous cancers were poorly reported. Assuming that all nine metachronous cancers in the intervention group were detected through colonoscopy, the NNS for detecting either recurrence or metachronous cancer was 106 (95% c.i. 73 to 160). Two trials^30,33^ specifically reported symptom status at the time of recurrence detection by colonoscopy. Among the 948 colonoscopies performed in these two studies, no asymptomatic intraluminal recurrences and only one asymptomatic metachronous cancer were identified.

### Time to recurrence

Four studies^29–32,34^ reported time to recurrence either as a mean^29^, median^30^ or using Kaplan–Meier curves^34^. One study^31,32^ provided a mean without an s.d., but also Kaplan–Meier curves; therefore, individual patient data were generated from the Kaplan–Meier curves. The results were harmonized and reported as MDs. A pooled subgroup analysis of patients with recurrence revealed that recurrence was detected approximately 5 months earlier in the intervention group (MD −5.14 months; 95% c.i. −7.25 to −3.04 months; *Fig. S3*).

### Curative-intent reoperations

Curative-intent reoperations for recurrent disease were reported in all trials, with higher rates in the intervention group (RR 1.66; 95% c.i. 1.16 to 2.37; *Fig. 3a*). Four trials reported curative-intent reresections for local recurrence^29–33^, suggesting a statistically non-significant trend favouring the intervention group (RR 2.20; 95% c.i. 0.97 to 4.97; *Fig. 3b*). Notably, the trials did not specify, which surveillance modality triggered the reoperation, nor did they report consistently whether an extra- or intraluminal recurrence was reoperated. Among metachronous cancers, four of five patients in the control group and eight of nine in the intervention group were operated on with curative intent.

**Figure 3:**
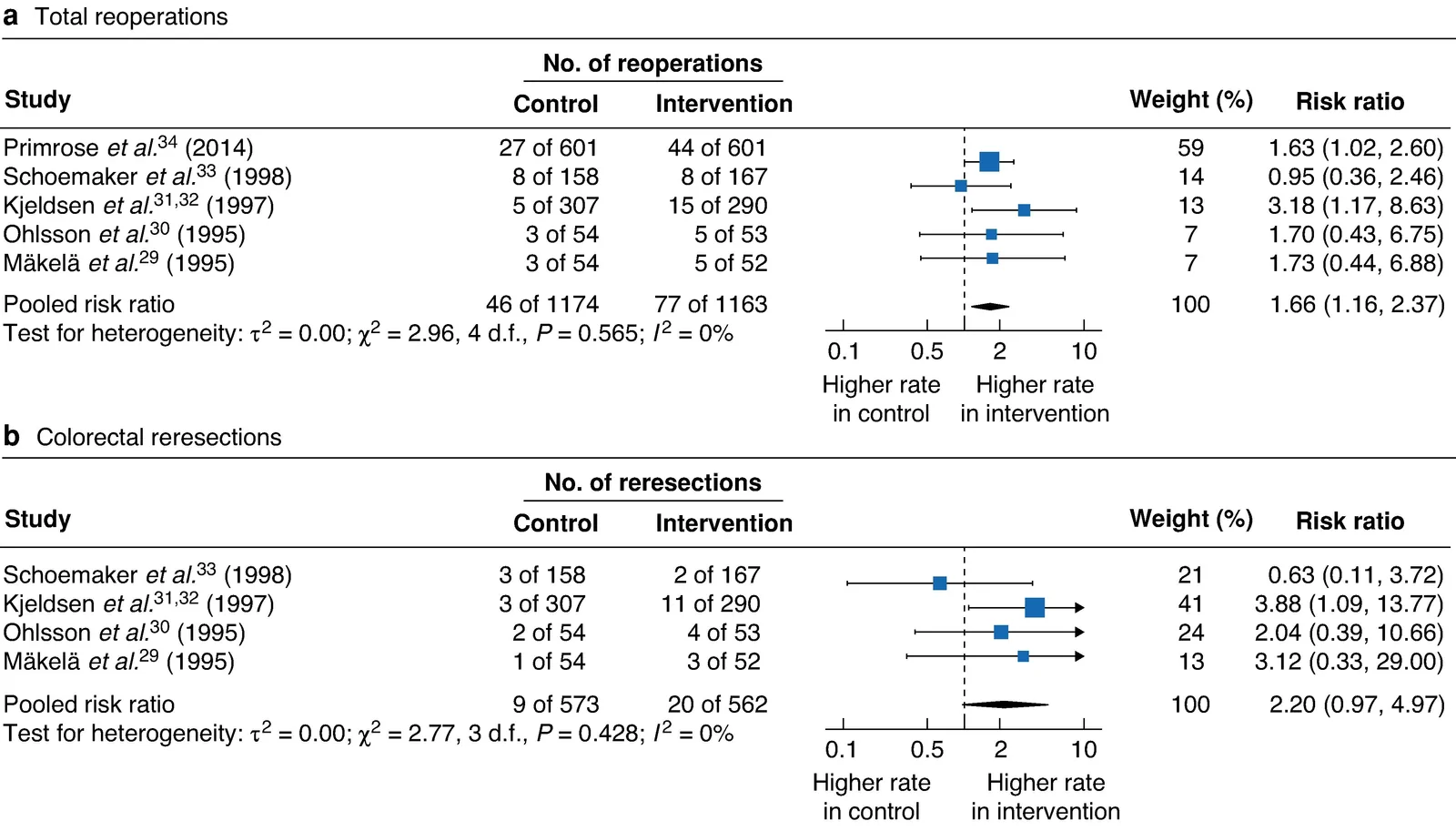
Reoperations during follow-up with protocols either including colonoscopy (intervention) or not (control) **a** Effects of the intervention on all reoperations. **b** Effects of the intervention on colorectal reresections. Values in parentheses are 95% confidence intervals. Effect sizes are presented as risk ratios using a random-effects model. Square size indicates weight. The numbers of reoperations in each group are shown, along with group size.

### Overall and cancer-specific mortality

All included trials reported overall mortality and the data required to calculate HRs. Across the studies, the intervention group experienced 277 all-cause deaths (23.8%), whereas the control group had 308 all-cause deaths (26.2%). Pooled analysis showed no significant difference in overall mortality between the groups (HR 0.92; 95% c.i. 0.77 to 1.11; *Fig. 4a*). Three trials^30–32,34^ reported cancer-specific mortality. One of these trials^34^ reported only crude cancer-specific death rates, which necessitated the use of RRs for pooled analysis. There was no significant difference in cancer-specific mortality between the groups (RR 0.97; 95% c.i. 0.78 to 1.21; *Fig. 4b*). Mortality related to intraluminal recurrence or metachronous cancer specifically was not reported.

**Figure 4:**
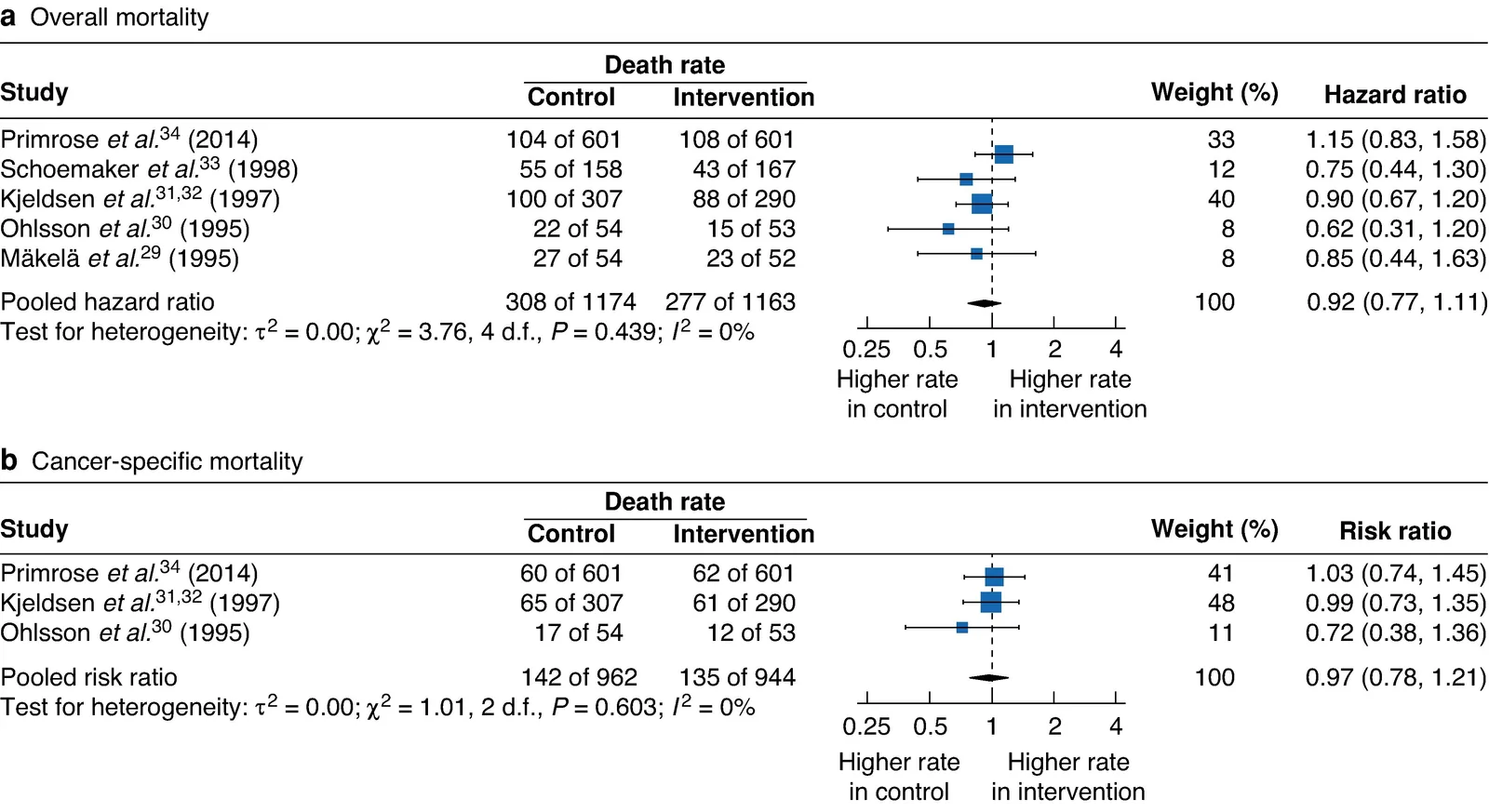
Mortality during follow-up with protocols either including colonoscopy (intervention) or not (control) **a** Effects of the intervention on overall mortality. **b** Effects of the intervention on cancer-specific mortality. Crude death rates are reported. Values in parentheses are 95% confidence intervals. Effect sizes are presented as hazard ratios for overall mortality and as risk ratios for cancer-specific mortality using a random-effects model. Square size indicates weight. The numbers of deaths in each group are shown, along with group size.

### Health economic considerations

Using National Health Service (NHS) pricing data, the cost of colonoscopy surveillance was estimated. According to the 2025–2026 NHS payment scheme, the cost of a diagnostic colonoscopy is £561 (∼€655)^35^. The NNS of 159 (95% c.i. 101 to 267) translates into a cost per colonoscopy-detected recurrence of approximately €104 100 (95% c.i. €66 200 to 174 900). Based on published data, curative-intent reresection for locally recurrent rectal cancer provides 1.35–1.92 QALYs^36^, which results in a cost of approximately €54 200–77 100 per QALY for colonoscopy-detected recurrences, assuming that all are resectable. This figure is restricted to diagnostic colonoscopy costs only and does not include costs of biopsy equipment, pathological analyses, clinic visits, complications, productivity losses, or treatment of the detected recurrence. Thus, the true costs are likely substantially higher. Moreover, the mean locoregional recurrence rate of 7.6% does not reflect the modern rates of 2–5%^8–10^. In this theoretical scenario, the NNS would be even higher, ranging from 242 to 604, assuming the colonoscopy detection rate and the total number of colonoscopies performed remain constant. These NNS figures correspond to a cost per colonoscopy-detected recurrence of €158 500–395 600 and a cost per QALY of €82 600–293 000.

### Quality of evidence assessment

Certainty of evidence was compromised primarily due to the confounding effect of multimodal surveillance strategies resulting in downgrading by one level in both risk of bias and indirectness across all outcomes. In addition, the evidence was further downgraded by one level for imprecision, because confidence intervals were either wide or often included both potential benefit and harm, indicating no difference. Overall, the certainty of evidence was judged to be low to very low (*Table S6*).

## Discussion

Five RCTs including more than 2300 patients who underwent surgery for CRC did not demonstrate a clear benefit of routine colonoscopy surveillance *versus* no colonoscopy surveillance within 5 years after CRC surgery. Follow-up protocols including colonoscopy surveillance led to a higher rate of detection of asymptomatic recurrences, approximately 5 months earlier, and a higher rate of curative-intent reoperations, without differences in overall or cancer-specific mortality.

An NNS of 159 is comparable to estimates from studies^37,38^ of screening colonoscopy without preceding faecal haemoglobin testing (NNS = 91–203), a strategy generally considered inefficient. Consistent with this, illustrative economic estimates based on colonoscopy costs alone suggested a cost of approximately €54 200–77 100 per QALY gained. Although these estimates exceed commonly cited UK willingness-to-pay thresholds of £20 000–30 000 (€23 000–35 000) per QALY^39^, they should be interpreted cautiously given the simplifying assumptions and absence of a formal incremental cost-effectiveness analysis. In a 2009 US decision-analytical modelling study^40^ using Medicare reimbursement data, a similar cost of US$40 313 (€55 611 after adjustment for inflation and currency) per life-year gained was estimated for performing an early colonoscopy.

The authors of the US study^40^ concluded that this figure was cost-effective when applying a threshold of US$150 000; notably, this exceeds substantially the corresponding UK threshold. In settings where endoscopy capacity is increasingly constrained, routine low-yield surveillance procedures also carry opportunity costs by diverting resources from higher-impact diagnostic and screening indications. Previous reviews^12,13^ reported survival benefit for colonoscopy, which has been one of the main arguments to justify colonoscopy surveillance^1^. Therefore, it is crucial that discrepancies with previous reviews are addressed with fidelity. The survival benefit for colonoscopy could not be reproduced in the present study, despite the higher number of pooled patients. This discrepancy may result from the study selection: one of the previous reviews^13^ did not clearly specify which studies were included in the subgroup analysis for colonoscopy surveillance, whereas the other^12^ included a study that actually investigated rigid sigmoidoscopy rather than colonoscopy^28^, and was therefore excluded in the present review. In addition, a thorough evaluation of the literature identified a further trial investigating colonoscopy surveillance^34^ that was not included in the earlier reviews. Finally, the survival benefit reported in previous reviews was not discussed comprehensively and the conclusions appeared selective; for example, Pita-Fernández *et al.*^13^ acknowledged that the observed survival benefit of chest X-ray may have been driven by other surveillance modalities under investigation, but did not draw the same conclusion for colonoscopy. By exclusively focusing on colonoscopy surveillance, the present review provides further insights into the topic; for example, the effectiveness of colonoscopy surveillance was further evaluated assessing NNS and health economic considerations.

The limitations of the present review largely reflect those of the included primary studies. First, a major limitation is that all trials investigated colonoscopy in combination with other surveillance modalities. From the perspective of this review (colonoscopy *versus* no colonoscopy), these represent unbalanced deviations from intended interventions (Domain 2 in RoB 2), even though from the trials’ own perspective (more *versus* less intensive follow-up), the risk of bias may not have been as high. Consequently, it cannot be concluded that any apparent benefits, in the present or previous reviews^12,13^, are attributable to colonoscopy.

Second, most trials were outdated. The total mesorectal excision technique for rectal cancer was introduced by Heald in the late 1980s and became standard of care from the mid-1990s to the early 2000s, resulting in a marked decline in locoregional recurrence^41^. Similarly, major advances in modern oncological therapy were made around the turn of the millennium with the transition from postoperative to preoperative radiotherapy in rectal cancer^42^ and the adoption of oxaliplatin-based chemotherapy regimens in CRC in general^43^. Four trials^29–33^ conducted in the 1980s and 1990s reported a pooled locoregional recurrence rate of 12.1% (range 6.5–21.7%). These figures are comparable to the rates before implementation of modern cancer therapies^5^, indicating that the evidence reflects outdated clinical practice. Because the efficiency of surveillance is closely tied to the frequency of the outcome, the substantially lower incidence of locoregional recurrence in more recent studies^8–10^ (∼2–5%) suggests even more reduced efficiency of colonoscopy surveillance in the modern era. In patients with colorectal or coloanal anastomoses, surveillance for intraluminal recurrence may be achievable with less invasive endoscopic approaches, questioning the need for routine full colonoscopy in this context. Third, the extracted data in this review rely on the values reported in the included studies, which required several assumptions. For example, HRs for survival were not provided and individual patient data were unavailable, which necessitated the reconstruction of outcomes from the Kaplan–Meier plots. In addition, variances in how time to recurrence was reported required harmonization across the studies, leading to potential inaccuracy. Calculation of health economic estimates was also based on several assumptions and should therefore be interpreted with caution. The authors acknowledge that the incremental cost-effectiveness ratio is the preferred measure in health economics. However, the costs of not conducting colonoscopies are negligible compared with repeated examinations but the effect is certainly more than zero. Thus, even though the incremental cost-effectiveness ratio is difficult to determine precisely, it could be even higher than these estimates. These estimates are not intended to define policy thresholds but to contextualize the clinical yield of colonoscopy surveillance relative to its procedural frequency and opportunity cost within constrained endoscopy services. Nevertheless, these exploratory estimates raise questions regarding the economic efficiency of routine colonoscopy surveillance and support the need for formal contemporary cost-effectiveness analyses.

Finally, the frequent inability of primary trials to distinguish intraluminal anastomotic recurrence from extraluminal locoregional disease is itself a critical limitation of the evidence base. If the effect of colonoscopy cannot be isolated at the trial level, it cannot be reliably recovered through meta-analysis.

Given the decreased risk of locoregional recurrence with modern treatment, the principal rationale for postoperative colonoscopy surveillance lies in the risk of metachronous cancer. There was no difference in metachronous cancer rates between the groups, but clinical outcomes related to metachronous cancer were poorly reported. Epidemiological data indicate an absolute annual risk of metachronous cancer between 0.63% and 0.74% during the first 3 years of follow-up, dropping thereafter to < 0.5%^44^. The temporal pattern likely reflects incompletely resected or missed synchronous lesions during the perioperative work-up rather than true early metachronous cancers. Instead of relying on postoperative colonoscopy surveillance to identify malignant lesions, emphasis should be placed on ensuring an adenoma-free colon with high-quality perioperative colonoscopy. Advances in endoscopic technology, including artificial intelligence assistance, have improved adenoma detection rates^45^, thereby further reducing the value of routine postoperative colonoscopy surveillance. Crucially, no randomized evidence demonstrates that detecting metachronous cancers within the first postoperative years through routine colonoscopy translates into improved overall or cancer-specific survival, particularly given the low absolute incidence and typically favourable stage at diagnosis.

It is acknowledged that postoperative surveillance recommendations are frequently developed in the context of imperfect evidence and must balance oncological vigilance, patient reassurance, and feasibility. Because colonoscopy is used to diagnose the index cancer, it has intuitive appeal as a low-mortality procedure capable of detecting intraluminal pathology. However, intuitive plausibility should not substitute for demonstrable clinical benefit when a practice is applied routinely, repeatedly, and at population scale. These findings should not be interpreted as an argument against postoperative colonoscopy surveillance *per se*, but as a call to re-examine which components of surveillance meaningfully influence outcomes. High-quality perioperative colonoscopy, along with imaging and carcinoembryonic antigen-based recurrence detection, may represent more rational targets for future surveillance strategies than routine short-interval colonoscopy.

Overall, current randomized evidence does not clearly demonstrate a survival benefit associated with routine postoperative colonoscopy surveillance. However, interpretation is limited by the age of the available trials, evolving treatment paradigms, and confounding from concurrent surveillance modalities. Consequently, the independent contribution of colonoscopy to contemporary surveillance strategies remains uncertain. A modern randomized trial designed to evaluate colonoscopy within current multimodal follow-up pathways is therefore warranted.

## Supplementary Material

zrag117_Supplementary_Data

## Data Availability

All data and the R code used in this study are available from the corresponding author upon reasonable request.
