# Supplementary material for: Colonoscopy surveillance after colorectal cancer surgery: meta-analysis
Source: BJS Open. 2026 Nov 4;10(6):zrag117. doi: 10.1093/bjsopen/zrag117 (PMC13732565; doi:10.1093/bjsopen/zrag117)
Supplement: Supplementary file 1 [file zrag117_supplementary_data.docx]

**Colonoscopy surveillance after colorectal cancer surgery: a systematic review and meta-analysis**

Joni Panula^1,2^, Kalle E Hokkanen^2^, Toni T Seppälä^1,2,3,4^

^1^Department of Gastroenterology and Alimentary Tract Surgery and Tays Cancer Centre, Tampere University Hospital, Wellbeing Services County of Pirkanmaa, Tampere, Finland

^2^Center for Cancer Eradication Research, Faculty of Medicine and Health Technology, Tampere

University, Tampere, Finland

^3^Applied Tumor Genomics, Research Programs Unit, University of Helsinki, Helsinki, Finland

^4^Abdominal Center, Helsinki University Central Hospital, HUS, Helsinki, Finlandation

**Corresponding author.**

Prof Toni T. Seppälä

Arvo Ylpön katu 34, 33520 Tampere, Finland

ORCID ID 0000-0002-4940-3498

**Supplementary Materials - Index**

| **Supplementary Figures and Tables** |  |
| --- | --- |
| Supplementary Table 1 | *page 2* |
| Supplementary Table 2 | *page 3* |
| Supplementary Table 3 | *page 4* |
| Supplementary Table 4 | *page 5-10* |
| Supplementary Table 5 | *page 11* |
| Supplementary Figure S1 | *page 12* |
| Supplementary Figure S2 | *page 13* |
| Supplementary Figure S3 | *page 14* |
| Supplementary Table 6  PRISMA 2020 Checklist | *page 15*  *page 16-18* |
| **References** | *page 19* |
|  |  |

**Supplementary Figures and Tables**

**Supplementary Table 1.** Search strategy

| Pubmed | (”Colorectal Neoplasms” [Mesh] OR (“colorectal cancer*” [tiab] OR “colorectal tumo*” [tiab] OR “colorectal neoplas*” [tiab] OR “colon cancer*” [tiab] OR “colon tumo*” [tiab] OR “colon neoplas*” [tiab] OR “rect* cancer*” [tiab] OR “rect* tumo*” [tiab] OR “rect* neoplas*” [tiab] OR “CRC” [tiab]))  AND (“surveillance” [tiab] OR “follow-up” [tiab] OR “follow up” [tiab])  AND (“postoperative” [tiab] OR “post-operative” [tiab] OR “after” [tiab] OR “post” [tiab])  AND ("colonoscopy" [Mesh] OR "colonoscop*" [All Fields] OR "endoscop*" [All Fields] OR "sigmoidoscop*" [All Fields])  AND (“randomized controlled trial” [pt] OR “controlled clinical trial” [pt] OR randomized [tiab] OR randomly [tiab] OR trial [tiab]) |
| --- | --- |
| Scopus | TITLE-ABS-KEY(("colorectal neoplasms" OR "colorectal cancer*" OR "colorectal tumo*" OR "colorectal neoplas*" OR "colon cancer*" OR "colon tumo*" OR "colon neoplas*" OR "rect* cancer*" OR "rect* tumo*" OR "rect* neoplas*" OR "CRC"))  AND TITLE-ABS-KEY(("surveillance" OR "follow-up" OR "follow up"))  AND TITLE-ABS-KEY(("postoperative" OR "post-operative" OR "after" OR "post"))  AND TITLE-ABS-KEY(("colonoscop*" OR "endoscop*" OR "sigmoidoscop*"))  AND TITLE-ABS-KEY(("randomized controlled trial" OR "controlled clinical trial" OR randomized OR randomly OR trial)) |
| Web of Science | TS=(("colorectal neoplasms" OR "colorectal cancer*" OR "colorectal tumo*" OR "colorectal neoplas*" OR "colon cancer*" OR "colon tumo*" OR "colon neoplas*" OR "rect* cancer*" OR "rect* tumo*" OR "rect* neoplas*" OR CRC))  AND TS=(surveillance OR "follow-up" OR "follow up")  AND TS=(postoperative OR "post-operative" OR after OR post)  AND TS=(colonoscop* OR endoscop* OR sigmoidoscop*)  AND TS=("randomized controlled trial" OR "controlled clinical trial" OR randomized OR randomly OR trial) |
| CENTRAL | (colorectal neoplasms OR colorectal cancer OR colorectal tumor OR colon cancer OR colon tumor OR rectal cancer OR rectal tumor OR CRC) AND (surveillance OR follow-up OR follow up) AND (postoperative OR post-operative OR after OR post) AND (colonoscopy OR colonoscope OR endoscopy OR sigmoidoscopy) in All Text |

**Supplementary Table 2.** Surveillance strategies in intervention and control groups.

|  | **Intervention group** | | **Control group** | |
| --- | --- | --- | --- | --- |
|  | **Colonoscopy** | **Other** | **Colonoscopy** | **Other** |
| Mäkelä et al.^1^ | Colonoscopy at 3 months if not performed preoperatively, and then once a year | US of the liver every 6 months, and CT of the liver and the site of the primary resection once a year  Flexible sigmoidoscopy every 3 months for rectal or sigmoid cancers | - | Barium enema once a year  Rigid sigmoidoscopy every 3 months for 2 years, and then every 6 months for next 3 years for rectal or sigmoid cancers |
| Ohlsson et al.^2^ | Complete colonoscopy at 3, 15, 30 and 60 months  Endoscopic control of the anastomosis at 9, 21 and 42 months | Physical examination, CEA, ALP, GGT, faecal Hb and CXR every 3 months first 2 years, every 6 months next 2 years, and then at 5 years  CT of the pelvis in patients undergoing APR at 3, 6, 12, 18 and 24 months  Rigid sigmoidoscopy every 3 months first 2 years, every 6 months next 2 years, and then at 5 years | - | Written instruction to leave faecal Hb every 3 months first 2 years, and then once a year, and to contact the surgical department if needed (problems with the colostomy, abdominal or perineal pain, altered bowel movements, change in faecal colour, micturition problems, or weight loss) |
| Kjeldsen et al.^3,4^ | Colonoscopy every 6 months for 3 years, once a year next 2 years, and then at 10, 12.5 and 15 years | Medical history, clinical examination, digital rectal examination, gynaecological examination, Haemoccult-II test, CXR, Hb level, ESR and liver enzymes at with the same intervals | Colonoscopy at 5, 10 and 15 years | Same examinations as in the intervention group, but at 5, 10 and 15 years |
| Schoemaker et al.^5^ | Colonoscopy once a year | CXR and CT of the liver once a year | Colonoscopy at 5 years to exclude undiagnosed recurrences and metastases | CXR and CT of the liver at 5 years to exclude undiagnosed recurrences and metastases |
| Primrose et al.^6^ | Colonoscopy at 2 years | CT of the chest, abdomen and pelvis every 6 months first 2 years, then once a year next 3 years  For a half of the group CEA every 3 months for first 2 years, then every 6 months next 3 years | - | No scheduled follow-up for a half of the group  For a half of the group CEA every 3 months for first 2 years, then every 6 months next 3 years  Single CT of the chest, abdomen and pelvis at 12-18 months was allowed if requested by hospital clinician |

Only examinations that were different or performed with different intervals are denoted in the table. Abbreviations: CT, computed tomography; US, ultrasonography; CXR, chest X-ray; ESR, erythrocyte sedimentation rate; Hb, haemoglobin; ALP, alkaline phosphatase; GGT, gamma-glutamyltransferase; APR, abdominoperineal resection.

**Supplementary Table 3.** Summary of risk of bias assessment

| **Study** | **Randomisation process** | **Deviations from intended intervention^1^** | **Missing outcome data** | **Measurement of outcome** | **Selection of the reported results** | **Overall** |
| --- | --- | --- | --- | --- | --- | --- |
| Mäkelä et al.^1^ | Some concerns | High | Low | Low | Some concerns | High |
| Ohlsson et al.^2^ | Some concerns | High | Low | Low | Some concerns | High |
| Kjeldsen et al.^3,4^ | Some concerns | High | Low | Low | Some concerns | High |
| Schoemaker et al.^5^ | High | High / Some concerns^2^ | Low | Low | Some concerns | High |
| Primrose et al.^6^ | Low | High | Low | Low | Low | High |

Footnotes: 1) Concurrent protocolized surveillance modalities were considered as unbalanced deviations from the intended intervention in relation to review’s aim. 2) Risk of bias was judged high for most outcomes with exception of intraluminal/locoregional recurrences and metachronous cancers.

**Supplementary Table 4.** Detailed risk of bias assessment

|  | | | | | **Domain 1. Randomization process** | | | | |
| --- | --- | --- | --- | --- | --- | --- | --- | --- | --- |
| **Study ID** | **Outcome** | **1.1 Was the allocation sequence random?** | **1.2 Was the allocation sequence concealed until participants were enrolled and assigned to interventions?** | **Note for 1.1&1.2** | | **1.3 Did baseline differences between intervention groups suggest a problem with the randomisation process?** | **Note for 1.3** | **1.0 Algorithm result** | **1.0 Assessor's Judgement** |
| Mäkelä 1995 | Group 1: Total, distant, and asymptomatic recurrences, time to recurrence, total curative-intent reoperations, curative-intenr re-resctions, overall survival, cancer-specific survival | Y | NI | "These patients were randomized during the hospitalization into two follow-up programs". No information was found on concealment. No difference was found in characteristics of participants in text, Figure 1 or Tables 1 and 2. | | N | No difference was found in characteristics of participants in text, Figure 1 or Tables 1 and 2. | Some concerns | **Some concerns** |
| Ohlsson 1995 | Group 1: Total, distant, and asymptomatic recurrences, time to recurrence, total curative-intent reoperations, curative-intenr re-resctions, overall survival, cancer-specific survival | Y | NI | "Patients were randomized to have no organized follow-up [...] or intense follow-up [...]."  No information was found on concealment. | | PN | Some difference in sex distribution (famele to male 23/31 and 33/20 in control and colonoscopy groups, respectively) but not in other characteristics in Table 2. | Some concerns | **Some concerns** |
| Kjeldsen 1997 | Group 1: Total, distant, and asymptomatic recurrences, time to recurrence, total curative-intent reoperations, curative-intenr re-resctions, overall survival, cancer-specific survival | Y | NI | "After surgery, the patients were allocated to one of two follow-up programmes (groups 1 and 2) by random numbers [...]". No information was found on concealment. | | N | No difference was found in characteristics of participants. | Some concerns | **Some concerns** |
| Schoemaker 1998 | Group 1: Total, distant, and asymptomatic recurrences, time to recurrence, total curative-intent reoperations, curative-intenr re-resctions, overall survival, cancer-specific survival | Y | PN | "The patients were then randomized to either standard or intensive follow-up by choosing the next card from a box of cards indicating the type of follow-up. The cards had been previously randomized using random tables." Cards seems not concealed. | | N | No difference was found in characteristics of participants in text or Table 1. | High | **High** |
| Primrose 2014 | Group 1: Total, distant, and asymptomatic recurrences, time to recurrence, total curative-intent reoperations, curative-intenr re-resctions, overall survival, cancer-specific survival | Y | Y | "Randomization [...] was performed centrally at the Oxford Clinical Trials Unit using a minimization algorithm [...]." | | N | No difference was found in characteristics of participants in text or Table 1. | Low | **Low** |
| Mäkelä 1995 | Group 2: Locoregional and intraluminal recurrences, and metachronous caners | Y | NI | "These patients were randomized during the hospitalization into two follow-up programs".  No information was found on concealment. | | N | No difference was found in characteristics of participants in text, Figure 1 or Tables 1 and 2. | Some concerns | **Some concerns** |
| Ohlsson 1995 | Group 2: Locoregional and intraluminal recurrences, and metachronous caners | Y | NI | "Patients were randomized to have no organized follow-up [...] or intense follow-up [...]."   No information was found on concealment. | | PN | Some difference in sex distribution (famele to male 23/31 and 33/20 in control and colonoscopy groups, respectively) but not in other characteristics in Table 2. | Some concerns | **Some concerns** |
| Kjeldsen 1997 | Group 2: Locoregional and intraluminal recurrences, and metachronous caners | Y | NI | "After surgery, the patients were allocated to one of two follow-up programmes (groups 1 and 2) by random numbers [...]".  No information was found on concealment. | | N | No difference was found in characteristics of participants in text or Table 1. | Some concerns | **Some concerns** |
| Schoemaker 1998 | Group 2: Locoregional and intraluminal recurrences, and metachronous caners | Y | PN | "The patients were then randomized to either standard or intensive follow-up by choosing the next card from a box of cards indicating the type of follow-up. The cards had been previously randomized using random tables."  Cards seems not concealed. | | N | No difference was found in characteristics of participants. | High | **High** |
| Primrose 2014 | Group 2: Locoregional and intraluminal recurrences, and metachronous caners | Y | Y | "Randomization [...] was performed centrally at the Oxford Clinical Trials Unit using a minimization algorithm [...]." | | N | No difference was found in characteristics of participants in text or Table 1. | Low | **Low** |

Abbreviations: Y, yes; PY, propably yes; N, no; PN, propably no; NI, no information; CT, computed tomography; US, ultrasound; CEA, carcinoembryonic antigen; Hb, haemoglobin; CXR, chest x-ray.

| **Domain 2. Deviations from intended interventions** | | | | | | |
| --- | --- | --- | --- | --- | --- | --- |
| **2.1 Were participants aware of their assigned intervention during the trial?** | **2.2 Were carers and people delivering the interventions aware of participants’ assigned intervention during the trial?** | **Note for 2.1&2.2** | **2.3 If Y/PY/NI to 2.1 or 2.2: Were there deviations from the intended intervention that arose because of the trial context?** | **Note for 2.3** | **2.4 If Y/PY/NI to 2.3: Were these deviations likely to have affected the outcome?** | **Note for 2.4** |
| Y | Y | The nature of the intervention, e.g. follow-up examinations, results in awareness. | Y | CT and liver US were protocolized, but for the estimand of the independent effect of colonoscopy these are deviations from the intended intervention. | Y | CT/US may detect recurrences early and potentially influence survival. |
| Y | Y | The nature of the intervention, e.g. follow-up examinations, results in awareness. | Y | CEA and faecal Hb among other modalities were protocolized, but for the estimand of the independent effect of colonoscopy these are deviations from the intended intervention. | Y | CEA and faecal Hb may detect recurrences early and potentially influence survival. |
| Y | Y | The nature of the intervention, e.g. follow-up examinations, results in awareness. | Y | Faecal Hb and CXR among other modalities were protocolized, but for the estimand of the independent effect of colonoscopy these are deviations from the intended intervention. | PY | Faecel Hb and CXR may detect recurrences early and potentially influence survival. |
| Y | Y | The nature of the intervention, e.g. follow-up examinations, results in awareness. | Y | CT of the liver and CXR were protocolized, but for the estimand of the independent effect of colonoscopy these are deviations from the intended intervention. | Y | Additional surveillance modalities may detect recurrences early and potentially influence survival. |
| Y | Y | The nature of the intervention, e.g. follow-up examinations, results in awareness. | Y | CT of the chest and abdomen was protocolized, but for the estimand of the independent effect of colonoscopy these are deviations from the intended intervention. | Y | CT may detect recurrences early and potentially influence survival. |
| Y | Y | The nature of the intervention, e.g. follow-up examinations, results in awareness. | Y | CT and liver US were protocolized, but for the estimand of the independent effect of colonoscopy these are deviations from the intended intervention. | Y | CT/US may detect recurrences early and potentially influence survival. |
| Y | Y | The nature of the intervention, e.g. follow-up examinations, results in awareness. | Y | CEA and faecal Hb among other modalities were protocolized, but for the estimand of the independent effect of colonoscopy these are deviations from the intended intervention. | Y | CEA and faecal Hb may detect recurrences early and potentially influence survival. |
| Y | Y | The nature of the intervention, e.g. follow-up examinations, results in awareness. | Y | Faecal Hb and CXR among other modalities were protocolized, but for the estimand of the independent effect of colonoscopy these are deviations from the intended intervention. | PY | Faecel Hb and CXR may detect recurrences early and potentially influence survival. |
| Y | Y | The nature of the intervention, e.g. follow-up examinations, results in awareness. | Y | CT of the liver and CXR were protocolized, but for the estimand of the independent effect of colonoscopy these are deviations from the intended intervention. | N | CT of the liver and CXR unlikely affect detection of locoregional/intraluminal recurrence or metachronous cancer |
| Y | Y | The nature of the intervention, e.g. follow-up examinations, results in awareness. | Y | CT of the chest and abdomen was protocolized, but for the estimand of the independent effect of colonoscopy these are deviations from the intended intervention. | Y | CT may detect recurrences early and potentially influence survival. |

| **Domain 2. Deviations from intended interventions** | | | | | | | |
| --- | --- | --- | --- | --- | --- | --- | --- |
| **2.5 If Y/PY to 2.4: Were these deviations from intended intervention balanced between groups?** | **Note for 2.5** | **2.6 Was an appropriate analysis used to estimate the effect of assignment to intervention?** | **Note for 2.6** | **2.7 If N/PN/NI to 2.6: Was there potential for a substantial impact (on the result) of the failure to analyse participants in the group to which they were randomised?** | **Note for 2.7** | **2.0 Algorithm result** | **2.0 Assessor's Judgement** |
| N | CT/US were protocolized for the intervention group only, so not balanced between groups. | Y | Intention-to-treat not reported, but all randomized participants appear included in analysis. | NA |  | High | **High** |
| N | Additional surveillance modalities were protocolized for the intervention group only, so not balanced between groups. | Y | Intention-to-treat not reported, but all randomized participants appear included in analysis. | NA |  | High | **High** |
| N | Additional surveillance modalities were protocolized for the intervention group only, so not balanced between groups. | PY | Intention-to-treat not reported, but all randomized participants appear included in analysis. | NA |  | High | **High** |
| N | Additional surveillance modalities were protocolized for the intervention group only, so not balanced between groups. | NI | Intention-to-treat not reported, any missing participant not reported. | PN |  | High | **High** |
| N | CT was protocolized for the intervention group only, so not balanced between groups. | Y | Intention-to-treat not reported for overall survival, but it was the prefered analysis and all randomized participants appear included in analysis. | NA |  | High | **High** |
| N | CT/US were protocolized for the intervention group only, so not balanced between groups. | Y | Intention-to-treat not reported, but all randomized participants appear included in analysis. | NA |  | High | **High** |
| N | Additional surveillance modalities were protocolized for the intervention group only, so not balanced between groups. | Y | Intention-to-treat not reported, but all randomized participants appear included in analysis. | NA |  | High | **High** |
| N | Additional surveillance modalities were protocolized for the intervention group only, so not balanced between groups. | PY | Intention-to-treat not reported, but all randomized participants appear included in analysis. | NA |  | High | **High** |
| NA |  | NI | Intention-to-treat not reported, any missing participant not reported. | PN |  | Some concerns | **Some concerns** |
| N | CT was protocolized for the intervention group only, so not balanced between groups. | Y | Intention-to-treat not reported for overall survival, but it was the prefered analysis and all randomized participants appear included in analysis. | NA |  | High | **High** |

| **Domain 3. Mising outcome data** | | | | | | | | | |
| --- | --- | --- | --- | --- | --- | --- | --- | --- | --- |
| **3.1 Were data for this outcome available for all, or nearly all, participants randomised?** | **Note for 3.1** | **3.2 If N/PN/NI to 3.1: Is there evidence that the result was not biased by missing outcome data?** | **Note for 3.2** | **3.3 If N/PN to 3.2: Could missingness in the outcome depend on its true value?** | **Note for 3.3&3.4** | **3.4 If Y/PY/NI to 3.3: Is it likely that missingness in the outcome depended on its true value?** | **Note for 3.4 (not use)** | **3.0 Algorithm result** | **3.0 Assessor's judgement** |
| Y | Kaplan-Meier curve includes all randomized participants; outcome data appear complete. | NA |  | NA |  | NA |  | Low | **Low** |
| Y | Kaplan-Meier curve includes all randomized participants; outcome data appear complete. | NA |  | NA |  | NA |  | Low | **Low** |
| Y | Kaplan-Meier curve includes all randomized participants; outcome data appear complete. | NA |  | NA |  | NA |  | Low | **Low** |
| PY | Participants in the survival analysis not reported, but there is no indications that participants were missing | NA |  | NA |  | NA |  | Low | **Low** |
| PY | Participants at risk table not reported with Kaplan-Meier curve, crude rates of death include all participants; outcome data seems complete. | NA |  | NA |  | NA |  | Low | **Low** |
| Y | Kaplan-Meier curve includes all randomized participants; outcome data appear complete. | NA |  | NA |  | NA |  | Low | **Low** |
| Y | Kaplan-Meier curve includes all randomized participants; outcome data appear complete. | NA |  | NA |  | NA |  | Low | **Low** |
| Y | Kaplan-Meier curve includes all randomized participants; outcome data appear complete. | NA |  | NA |  | NA |  | Low | **Low** |
| PY | Participants in the survival analysis not reported, but there is no indications that participants were missing | NA |  | NA |  | NA |  | Low | **Low** |
| PY | Participants at risk table not reported with Kaplan-Meier curve, crude rates of death include all participants; outcome data seems complete. | NA |  | NA |  | NA |  | Low | **Low** |

| **Domain 4. Measurement of the outcome** | | | | | | | | | | | |
| --- | --- | --- | --- | --- | --- | --- | --- | --- | --- | --- | --- |
| **4.1 Was the method of measuring the outcome inappropriate?** | **Note for 4.1** | **4.2 Could measurement or ascertainment of the outcome have differed between intervention groups?** | **Note for 4.2** | **4.3 If N/PN/NI to 4.1 and 4.2: Were outcome assessors aware of the intervention received by study participants?** | **Note for 4.3** | **4.4 If Y/PY/NI to 4.3: Could assessment of the outcome have been influenced by knowledge of intervention received?** | **Note for 4.4&4.5** | **4.5 If Y/PY/NI to 4.4: Is it likely that assessment of the outcome was influenced by knowledge of intervention received?** | **Note for 4.5 (not use)** | **4.0 Algorithm result** | **4.0 Assessor's Judgement** |
| N | Outcome measured using standard clinical methods (Kaplan-Meier survival), considered appropriate. | N | Survival measured objectively; ascertainment not influenced by intervention. | PY | Most likely, outcome assessors were aware of the surveillance strategy similarly to carers. | N | Assessment of survival is unlikely to be affected by the awareness. | NA |  | Low | **Low** |
| N | Outcome measured using standard clinical methods (Kaplan-Meier survival), considered appropriate. | N | Survival measured objectively; ascertainment not influenced by intervention. | PY | Most likely, outcome assessors were aware of the surveillance strategy similarly to carers. | N | Assessment of survival is unlikely to be affected by the awareness. | NA |  | Low | **Low** |
| N | Outcome measured using standard clinical methods (Kaplan-Meier survival), considered appropriate. | N | Survival measured objectively; ascertainment not influenced by intervention. | PY | Most likely, outcome assessors were aware of the surveillance strategy similarly to carers. | N | Assessment of survival is unlikely to be affected by the awareness. | NA |  | Low | **Low** |
| N | Outcome measured using standard clinical methods (Kaplan-Meier survival), considered appropriate. | N | Survival measured objectively; ascertainment not influenced by intervention. | PY | Most likely, outcome assessors were aware of the surveillance strategy similarly to carers. | N | Assessment of survival is unlikely to be affected by the awareness. | NA |  | Low | **Low** |
| N | Outcome measured using standard clinical methods (Kaplan-Meier survival), considered appropriate. | N | Survival measured objectively; ascertainment not influenced by intervention. | N | "However, the research staff who abstracted outcome data from clinical notes were employed by the local National Cancer Research Network teams independent of the investigators." | NA |  | NA |  | Low | **Low** |
| N | Outcome measured using standard clinical methods (Kaplan-Meier survival), considered appropriate. | N | Survival measured objectively; ascertainment not influenced by intervention. | PY | Most likely, outcome assessors were aware of the surveillance strategy similarly to carers. | N | Assessment of survival is unlikely to be affected by the awareness. | NA |  | Low | **Low** |
| N | Outcome measured using standard clinical methods (Kaplan-Meier survival), considered appropriate. | N | Survival measured objectively; ascertainment not influenced by intervention. | PY | Most likely, outcome assessors were aware of the surveillance strategy similarly to carers. | N | Assessment of survival is unlikely to be affected by the awareness. | NA |  | Low | **Low** |
| N | Outcome measured using standard clinical methods (Kaplan-Meier survival), considered appropriate. | N | Survival measured objectively; ascertainment not influenced by intervention. | PY | Most likely, outcome assessors were aware of the surveillance strategy similarly to carers. | N | Assessment of survival is unlikely to be affected by the awareness. | NA |  | Low | **Low** |
| N | Outcome measured using standard clinical methods (Kaplan-Meier survival), considered appropriate. | N | Survival measured objectively; ascertainment not influenced by intervention. | PY | Most likely, outcome assessors were aware of the surveillance strategy similarly to carers. | N | Assessment of survival is unlikely to be affected by the awareness. | NA |  | Low | **Low** |
| N | Outcome measured using standard clinical methods (Kaplan-Meier survival), considered appropriate. | N | Survival measured objectively; ascertainment not influenced by intervention. | N | "However, the research staff who abstracted outcome data from clinical notes were employed by the local National Cancer Research Network teams independent of the investigators." | NA |  | NA |  | Low | **Low** |

| **Domain 5. Selection of the reported result** | | | | | | | | **Domain 6. Overall Bias** | |
| --- | --- | --- | --- | --- | --- | --- | --- | --- | --- |
| **5.1 Were the data that produced this result analysed in accordance with a prespecified analysis plan that was finalised before unblinded outcome data were available for analysis?** | **Note for 5.1** | **5.2 Is the numerical result being assessed likely to have been selected, on the basis of the results, from multiple eligible outcome measurements (eg, scales, definitions, time points) within the outcome domain?** | **Note for 5.2** | **5.3 Is the numerical result being assessed likely to have been selected, on the basis of the results, from multiple eligible analyses of the data?** | **Note for 5.3** | **5.0 Algorithm result** | **5.0 Assessor's Judgement** | **Algorithm's overall Judgement** | **Assessor's overall Judgement** |
| NI | No protocol or pre-specified analysis plan reported; cannot verify outcome reporting. | N | Only Kaplan-Meier curve reported; no alternative effect measures presented. | N | Only Kaplan-Meier curve reported; no alternative analyses presented. | Some concerns | **Some concerns** | High | **High** |
| NI | No protocol or pre-specified analysis plan reported; cannot verify outcome reporting. | N | Only Kaplan-Meier curve reported; no alternative effect measures presented. | N | Only Kaplan-Meier curve reported; no alternative analyses presented. | Some concerns | **Some concerns** | High | **High** |
| NI | No protocol or pre-specified analysis plan reported; cannot verify outcome reporting. | N | Only Kaplan-Meier curve reported; no alternative effect measures presented. | N | Only Kaplan-Meier curve reported; no alternative analyses presented. | Some concerns | **Some concerns** | High | **High** |
| NI | No protocol or pre-specified analysis plan reported; cannot verify outcome reporting. | N | Only Kaplan-Meier curve reported; no alternative effect measures presented. | N | Only Kaplan-Meier curve reported; no alternative analyses presented. | Some concerns | **Some concerns** | High | **High** |
| PY | Study registered in clinicaltrials.gov and isrctn.com. | N | Only Kaplan-Meier curve reported; no alternative effect measures presented. | N | Only Kaplan-Meier curve reported; no alternative analyses presented. | Low | **Low** | High | **High** |
| NI | No protocol or pre-specified analysis plan reported; cannot verify outcome reporting. | N | Only Kaplan-Meier curve reported; no alternative effect measures presented. | N | Only Kaplan-Meier curve reported; no alternative analyses presented. | Some concerns | **Some concerns** | High | **High** |
| NI | No protocol or pre-specified analysis plan reported; cannot verify outcome reporting. | N | Only Kaplan-Meier curve reported; no alternative effect measures presented. | N | Only Kaplan-Meier curve reported; no alternative analyses presented. | Some concerns | **Some concerns** | High | **High** |
| NI | No protocol or pre-specified analysis plan reported; cannot verify outcome reporting. | N | Only Kaplan-Meier curve reported; no alternative effect measures presented. | N | Only Kaplan-Meier curve reported; no alternative analyses presented. | Some concerns | **Some concerns** | High | **High** |
| NI | No protocol or pre-specified analysis plan reported; cannot verify outcome reporting. | N | Only Kaplan-Meier curve reported; no alternative effect measures presented. | N | Only Kaplan-Meier curve reported; no alternative analyses presented. | Some concerns | **Some concerns** | High | **High** |
| PY | Study registered in clinicaltrials.gov and isrctn.com. | N | Only Kaplan-Meier curve reported; no alternative effect measures presented. | N | Only Kaplan-Meier curve reported; no alternative analyses presented. | Low | **Low** | High | **High** |

**Supplementary Table 5.** Recurrence and metachronous cancer rates.

| **Study** | **Year** | **Patients, total** | **Locoregional recurrences** | **Locoregional recurrence rate** | **Distant recurrences** | **Distant recurrence rate** | **Metachronous cancers** | **Metachronous cancer rate** |
| --- | --- | --- | --- | --- | --- | --- | --- | --- |
| Mäkelä et al.^1^ | 1995 | 106 | 23 | 21.7% | 20 | 18.9% | 1 | 0.9% |
| Ohlsson et al.^2^ | 1995 | 107 | 19 | 17.8% | 20 | 18.7% | 1 | 0.9% |
| Kjeldsen et al.^3,4^ | 1997 | 597 | 74 | 12.4% | 82 | 13.7% | 10 | 1.7% |
| Schoemaker et al.^5^ | 1998 | 325 | 21 | 6.5% | 51 | 15.7% | 2 | 0.6% |
| Primrose et al.^6^ | 2014 | 1202 | 41 | 3.4% | 158 | 13.1% | 3 | 0.2% |

**Supplementary Figure S1.** Asymptomatic recurrence rates in follow-up protocol including colonoscopy (intervention) vs. without colonoscopy (control) omitting Schoemaker et al 33. Effect sizes are presented as risk ratios using a random-effects model. Square size indicates weight. n = asymptomatic recurrences, N = group size.

**Supplementary Figure S2.** Metachronous cancer rates in follow-up protocol including colonoscopy (intervention) vs. without colonoscopy (control). Effect sizes are presented as risk ratios using a random-effects model. Square size indicates weight. n = metachronous cancers, N = group size.

**Supplemental Figure S3.** Time to recurrence (months) in follow-up protocol including colonoscopy (intervention) vs. without colonoscopy (control). Individual study results are presented in months with their standard deviation in parentheses. Effect sizes are presented as mean difference using a random-effects model. Square size indicates weight.

**Supplementary Table 6.** GRADE evidence profile.

|  |  | **Absolute effect** | |  |  |
| --- | --- | --- | --- | --- | --- |
| **Outcome** | **No. of patients (studies)** | **Intervention** | **Control** | **Relative effect (95% CI)** | **Certainty (GRADE)** |
| Intraluminal recurrences | N = 538 (3 studies) | 13 of 272 | 16 of 266 | RR = 0·78 (0·38–1·59) | Very low^1,2,3^ |
| Locoregional recurrences | N = 2337 (5 studies) | 97 of 1163 | 81 of 1174 | RR = 1·23 (0·93–1·63) | Very low^1,2,3^ |
| Asymptomatic recurrences | N = 2337 (5 studies) | 170 of 1163 | 73 of 1174 | RR = 2·38 (1·53–3·69) | Low^1,3^ |
| Curative-intent reoperations | N = 2337 (5 studies) | 77 of 1163 | 46 of 1174 | RR = 1·66 (1·16–2·37) | Low^1,3^ |
| Overall mortality | N = 2337 (5 studies) | 277 of 1163 | 308 of 1174 | HR = 0·92 (0·77–1·11) | Very low^1,2,3^ |

Footnotes: 1) Downgraded one level for risk of bias: all included trials evaluated colonoscopy as part of multimodal surveillance, preventing attribution of effects to colonoscopy alone; 2) Downgraded one level for imprecision: Confidence intervals are wide and/or include both benefit and harm; 3) Downgraded one level for indirectness: Same reason as Footnote 1 (multimodal interventions).

| **Section and Topic** | **Item #** | **Checklist item** | **Location where item is reported** |
| --- | --- | --- | --- |
| **TITLE** | | |  |
| Title | 1 | Identify the report as a systematic review. | Title |
| **ABSTRACT** | | |  |
| Abstract | 2 | See the PRISMA 2020 for Abstracts checklist. | The PRISMA abstract checklist used |
| **INTRODUCTION** | | |  |
| Rationale | 3 | Describe the rationale for the review in the context of existing knowledge. | Introduction |
| Objectives | 4 | Provide an explicit statement of the objective(s) or question(s) the review addresses. | End of Introduction |
| **METHODS** | | |  |
| Eligibility criteria | 5 | Specify the inclusion and exclusion criteria for the review and how studies were grouped for the syntheses. | Methods; Eligibility criteria |
| Information sources | 6 | Specify all databases, registers, websites, organisations, reference lists and other sources searched or consulted to identify studies. Specify the date when each source was last searched or consulted. | Methods; Search strategy |
| Search strategy | 7 | Present the full search strategies for all databases, registers and websites, including any filters and limits used. | Suppl. table 1 |
| Selection process | 8 | Specify the methods used to decide whether a study met the inclusion criteria of the review, including how many reviewers screened each record and each report retrieved, whether they worked independently, and if applicable, details of automation tools used in the process. | Methods; Study selection |
| Data collection process | 9 | Specify the methods used to collect data from reports, including how many reviewers collected data from each report, whether they worked independently, any processes for obtaining or confirming data from study investigators, and if applicable, details of automation tools used in the process. | Methods; Data analysis |
| Data items | 10a | List and define all outcomes for which data were sought. Specify whether all results that were compatible with each outcome domain in each study were sought (e.g. for all measures, time points, analyses), and if not, the methods used to decide which results to collect. | Methods; Data analysis |
|  | 10b | List and define all other variables for which data were sought (e.g. participant and intervention characteristics, funding sources). Describe any assumptions made about any missing or unclear information. | Methods; Data analysis |
| Study risk of bias assessment | 11 | Specify the methods used to assess risk of bias in the included studies, including details of the tool(s) used, how many reviewers assessed each study and whether they worked independently, and if applicable, details of automation tools used in the process. | Methods; Risk of bias assessment |
| Effect measures | 12 | Specify for each outcome the effect measure(s) (e.g. risk ratio, mean difference) used in the synthesis or presentation of results. | Methods; Data synthesis and statistical analysis |
| Synthesis methods | 13a | Describe the processes used to decide which studies were eligible for each synthesis (e.g. tabulating the study intervention characteristics and comparing against the planned groups for each synthesis (item #5)). | Not applicable* |
|  | 13b | Describe any methods required to prepare the data for presentation or synthesis, such as handling of missing summary statistics, or data conversions. | Methods; Data synthesis and statistical analysis |
|  | 13c | Describe any methods used to tabulate or visually display results of individual studies and syntheses. | Methods; Data synthesis and statistical analysis |
|  | 13d | Describe any methods used to synthesize results and provide a rationale for the choice(s). If meta-analysis was performed, describe the model(s), method(s) to identify the presence and extent of statistical heterogeneity, and software package(s) used. | Methods; Data synthesis and statistical analysis |
|  | 13e | Describe any methods used to explore possible causes of heterogeneity among study results (e.g. subgroup analysis, meta-regression). | Not applicable** |
|  | 13f | Describe any sensitivity analyses conducted to assess robustness of the synthesized results. | Not applicable*** |
| Reporting bias assessment | 14 | Describe any methods used to assess risk of bias due to missing results in a synthesis (arising from reporting biases). | Not applicable**** |
| Certainty assessment | 15 | Describe any methods used to assess certainty (or confidence) in the body of evidence for an outcome. | Methods; Certainty of evidence assessment |
| **RESULTS** | | |  |
| Study selection | 16a | Describe the results of the search and selection process, from the number of records identified in the search to the number of studies included in the review, ideally using a flow diagram. | Results; Study selection |
|  | 16b | Cite studies that might appear to meet the inclusion criteria, but which were excluded, and explain why they were excluded. | Results; Study selection |
| Study characteristics | 17 | Cite each included study and present its characteristics. | Results; Study characteristics, Table 1, Suppl table 2 |
| Risk of bias in studies | 18 | Present assessments of risk of bias for each included study. | Results; Risk of bias in studies, Suppl tables 3-4 |
| Results of individual studies | 19 | For all outcomes, present, for each study: (a) summary statistics for each group (where appropriate) and (b) an effect estimate and its precision (e.g. confidence/credible interval), ideally using structured tables or plots. | Figures 1-4, Suppl figs 1-3 |
| Results of syntheses | 20a | For each synthesis, briefly summarise the characteristics and risk of bias among contributing studies. | Suppl material 3-4 (RoB) and each outcome section |
|  | 20b | Present results of all statistical syntheses conducted. If meta-analysis was done, present for each the summary estimate and its precision (e.g. confidence/credible interval) and measures of statistical heterogeneity. If comparing groups, describe the direction of the effect. | Each outcome section |
|  | 20c | Present results of all investigations of possible causes of heterogeneity among study results. | Not applicable** |
|  | 20d | Present results of all sensitivity analyses conducted to assess the robustness of the synthesized results. | Not applicable*** |
| Reporting biases | 21 | Present assessments of risk of bias due to missing results (arising from reporting biases) for each synthesis assessed. | Not applicable**** |
| Certainty of evidence | 22 | Present assessments of certainty (or confidence) in the body of evidence for each outcome assessed. | Results; Quality of evidence assessment, Suppl material 6 |
| **DISCUSSION** | | |  |
| Discussion | 23a | Provide a general interpretation of the results in the context of other evidence. | Discussion; first third |
|  | 23b | Discuss any limitations of the evidence included in the review. | Discussion; second third |
|  | 23c | Discuss any limitations of the review processes used. | Discussion; second third |
|  | 23d | Discuss implications of the results for practice, policy, and future research. | Discussion; last third |
| **OTHER INFORMATION** | | |  |
| Registration and protocol | 24a | Provide registration information for the review, including register name and registration number, or state that the review was not registered. | Methods; Study design and systematic review registration |
|  | 24b | Indicate where the review protocol can be accessed, or state that a protocol was not prepared. | Methods; Study design and systematic review registration |
|  | 24c | Describe and explain any amendments to information provided at registration or in the protocol. | In PROSPERO |
| Support | 25 | Describe sources of financial or non-financial support for the review, and the role of the funders or sponsors in the review. | Funding |
| Competing interests | 26 | Declare any competing interests of review authors. | Declaration of interest, ICMJE forms |
| Availability of data, code and other materials | 27 | Report which of the following are publicly available and where they can be found: template data collection forms; data extracted from included studies; data used for all analyses; analytic code; any other materials used in the review. | Data sharing |

*From:* Page MJ, McKenzie JE, Bossuyt PM, Boutron I, Hoffmann TC, Mulrow CD, et al. The PRISMA 2020 statement: an updated guideline for reporting systematic reviews. BMJ 2021;372:n71. doi: 10.1136/bmj.n71. This work is licensed under CC BY 4.0. To view a copy of this license, visit <https://creativecommons.org/licenses/by/4.0/>

*) No subgroup analyses or separate synthesis groups were planned due to the limited number of studies and comparable intervention characteristics. Therefore, all studies were considered eligible for each synthesis provided they reported the corresponding outcome.

**) As heterogeneity was low, no subgroup analyses or meta-regression were performed to explore potential causes of heterogeneity

***) No sensitivity analyses were conducted, as the limited number of included studies and consistent findings did not allow robust exploration of alternative analytical assumptions.

****) No assessment for reporting bias was conducted due to the limited number of studies (n = 5), as specified in the study protocol (< 10)

**References**

1.	Mäkelä JT, Laitinen SO, Kairaluoma MI. Five-year follow-up after radical surgery for colorectal cancer. Results of a prospective randomized trial. Arch Surg Chic Ill 1960. 1995 Oct;130(10):1062–7. doi:10.1001/archsurg.1995.01430100040009 PubMed PMID: 7575117.

2.	Ohlsson B, Breland U, Ekberg H, Graffner H, Tranberg KG. Follow-up after curative surgery for colorectal carcinoma. Randomized comparison with no follow-up. Dis Colon Rectum. 1995 Jun;38(6):619–26. doi:10.1007/BF02054122 PubMed PMID: 7774474.

3.	Kjeldsen BJ, Kronborg O, Fenger C, Jørgensen OD. A prospective randomized study of follow-up after radical surgery for colorectal cancer. Br J Surg. 1997 May;84(5):666–9. PubMed PMID: 9171758.

4.	Kjeldsen BJ, Kronborg O, Fenger C, Jørgensen OD. The pattern of recurrent colorectal cancer in a prospective randomised study and the characteristics of diagnostic tests. Int J Colorectal Dis. 1997;12(6):329–34. doi:10.1007/s003840050118 PubMed PMID: 9457525.

5.	Schoemaker D, Black R, Giles L, Toouli J. Yearly colonoscopy, liver CT, and chest radiography do not influence 5-year survival of colorectal cancer patients. Gastroenterology. 1998 Jan;114(1):7–14. doi:10.1016/s0016-5085(98)70626-2 PubMed PMID: 9428212.

6.	Primrose JN, Perera R, Gray A, Rose P, Fuller A, Corkhill A, et al. Effect of 3 to 5 years of scheduled CEA and CT follow-up to detect recurrence of colorectal cancer: the FACS randomized clinical trial. JAMA. 2014 Jan 15;311(3):263–70. doi:10.1001/jama.2013.285718 PubMed PMID: 24430319.
